# Structural Knowledge Is What Matters in Protein–Ligand Binding Affinity Prediction

**DOI:** 10.3390/molecules31122025

**Published:** 2026-06-10

**Authors:** Natàlia Segura-Alabart, Francesc Serratosa

**Affiliations:** Departament d’Enginyeria Informàtica i Mateàtiques, Universitat Rovira i Virgili, 43007 Tarragona, Spain; natalia.segura@urv.cat

**Keywords:** binding affinity prediction, attributed graph, Graph Neural Networks, *pI*C50, PDBBind 2016, CASF 2016

## Abstract

Binding affinity prediction is about estimating the degree to which a drug binds to a protein. Predicting the binding affinity between a drug and a protein in a computational process helps researchers filter huge libraries of compounds before performing expensive biochemical lab experiments. Currently, there is interest in predicting binding affinity through computational pattern recognition or machine learning methods instead of the classical physics-inspired methods, which are computationally intractable except for tiny chemical compounds. In the last five years, several machine learning-based methods have been presented, whose experimental validations have achieved increasing Pearson coefficients while trained and tested in the PDBBind 2016 and CASF 2016 databases, respectively. These methods have an important diversity of architectures that provide different properties. The aim of this paper is to discern which binary properties (existence or absence) of these methods make them return higher Pearson coefficients. Basically, the properties introduced are related to the level of structural knowledge, the presence of 3D information, and the introduction of the relationship between the drug and the protein in the input of the model. The *t*-test confirms that the important binary properties for having a high Pearson coefficient are the protein (or part of the protein) being represented and introduced into the computational model as a graph, the pocket and the drug–protein interaction being part of the input, and incorporating the distance between atoms and the type of chemical bonds into the model.

## 1. Introduction

Binding affinity prediction is the computational process of estimating how strongly two molecules interact with each other, most commonly a drug (also called a ligand) and a biological target (usually a protein, DNA or RNA). In drug discovery, a chemical compound that acts as a drug that binds strongly (high affinity) to a protein involved in disease can block or activate the function of that protein, making it a potential medicine.

Predicting the binding affinity in a computational process helps researchers filter large libraries of compounds before doing expensive biochemical experiments [1]. Computational methods for binding affinity prediction can be classified into two categories. The first category consists of physics-inspired methods, which rely on fundamental physics principles such as molecular mechanics, thermodynamics, or quantum mechanics. These approaches have the strength of being grounded in well-established theory; however, their primary limitation is their accuracy, considering their substantial computational cost.

The second category of methods is the ones that are based on machine learning. These methods learn predictive patterns from large datasets and can be divided into two subgroups. The first subgroup includes classical machine learning approaches such as Random Forests, Support Vector Machines, Gradient Boosted Trees, and linear regression models. These methods typically rely on engineered input features, including molecular fingerprints, physicochemical descriptors, docking scores, or interaction terms.

In contrast, the second subgroup consists of deep learning approaches, which use structural information (3D) more directly through task-specific learning. For example, some models use Convolutional Neural Networks on 3D protein–ligand grids, while others use Graph Neural Networks that represent proteins and ligands as graphs [2,3]. Deep learning methods offer advantages such as scalability, rapid prediction once trained, and the ability to capture complex and non-linear patterns. Nevertheless, their weakness is that they require large amounts of high-quality training data; otherwise, they may fail to generalize outside the training domain.

To interpret the outputs of these computational methods, it is essential to understand how the binding affinity is quantitatively expressed. Binding affinity can be expressed in terms of constants such as $K_{d}$ (dissociation constant) or $K_{i}$ (inhibition constant). The lower $K_{d}$ or $K_{i}$ represents a stronger binding affinity. It may be also expressed as $IC_{50}$, which expresses the half-maximum inhibitory concentration. This is the concentration of a substance required to inhibit a given biological process or target by 50%, usually expressed in molar units [4]. Again, a lower $IC_{50}$ represents a stronger binding affinity. The relation between $K_{i}$ and $IC_{50}$ is modelled through the Cheng–Prusoff equation [5]. Finally, in computational chemistry, a common way to measure the binding affinity is by the pIC50, which is the negative logarithm of $IC_{50}$ and has no units. The advantage of using pIC50 instead of $IC_{50}$ is that the logarithm compresses the range into manageable numbers, and it has been demonstrated to have better statistical behaviour. Moreover, pIC50 is more interpretable than ${IC}_{50}$. For instance, pIC50 = 8 is ten times more potent than pIC50 = 7.

The PDBbind is a widely used benchmark dataset in computational chemistry, bioinformatics, and drug discovery [6]. It contains protein–ligand complexes that are deposited in the Protein Data Bank with their 3D structure and the experimentally measured values $K_{d}$, $K_{i}$ and $IC_{50}$. Computational models are trained using the protein–ligand structures and the pIC50 calculated through the $IC_{50}$.

The purpose of this paper is twofold. We first present a taxonomy of the latest deep learning-based methods for binding affinity prediction. Fifty different methods have been selected from the literature and classified according to 8 binary properties. These methods were then sorted using the Pearson correlation coefficient obtained when trained on the PDBbind 2016 dataset and tested on the CASF 2016 benchmark [7]. Based on the eight binary categories and their corresponding Pearson coefficients, a statistical analysis was done to deduce which properties were the most important to achieve the best Pearson coefficient. Although an important review of these 50 methods has been carried out to extract these eight properties, this paper is not presented as a review, such as [8,9], but an analysis of the importance of introducing structural knowledge in the computational protein–ligand binding affinity prediction.

The remainder of this paper is organised as follows. First, in Section 4.1, we define the binding affinity and briefly describe both old and new computational methods. Section 4.2 presents the experimental performance metrics reported for several binding affinity prediction methods across the literature. Section 4.3 describes the structural properties that commonly appear in these methods and a list of the methods reported that have these properties. Section 2.1 analyses, through a Student’s *t*-test, which of the properties that have been identified in Section 4.3 have more influence on the Pearson coefficients obtained by the predictive methods discussed in Section 4.2. Finally, Section 2.2 analyses, through a Chi-square test, which properties are dependent on each other. There are two final sections, with a discussion of the results and conclusions.

## 2. Results

The aim of the experiments is to correlate the information shown in Table 5 (structural knowledge) with that shown in Table 4 (Pearson coefficient). The aim is to find which binary properties have the most influence on having a high Pearson coefficient. In the first step, an analysis of the importance of each variable is conducted through a hypothesis test of means, to move in the second step to the analysis of the independence of variables through the Chi-square test.

### 2.1. Analysis of the Importance of Variables

The hypothesis test of means is a statistical test that can be used to discern two hypotheses. The first hypothesis, hypothesis $H_{A}$, assumes that the mean $m_{0}$ of population $P_{0}$ is equal to the mean $m_{1}$ of another population, $P_{1}$. In contrast, hypothesis $H_{B}$ assumes that $m_{0}$ < $m_{1}$.

In our case, the hypothesis test of means has been used to verify whether a binary property is useful in giving a model a high Pearson coefficient—that is, if, in general, the models that have this property tend to have a larger Pearson coefficient than the ones that do not have this property.

Eight different populations $P_{1}\left(1\right)$, …, $P_{1}\left(8\right)$, each one $P_{1}\left(i\right)$, 1≤i≤8, are composed of the Pearson coefficients of the models that have a 1 in one *i*th binary property. Note that these populations are not disjointed, since the Pearson coefficient of a specific model appears in all populations $P_{1}\left(i\right)$ that have a 1 in the *i*th column in Table 5. Similarly, $P_{0}\left(i\right)$, 1≤i≤8, is composed of the Pearson coefficients of the models that have a 0 in one *i*th binary property.

Thus, in our case, hypothesis $H_{A}\left(i\right)$, 1≤i≤8, assumes that property *i*th does not influence the Pearson coefficient since the mean of the values whose models do not have property *i*th is equal to the mean of the values whose models have property *i*th, that is, $m_{0}\left(i\right)$ = $m_{1}\left(i\right)$. In contrast, hypothesis $H_{B}\left(i\right)$ proposes that the *i*th property does influence the Pearson coefficient, specifically by increasing it, which corresponds to $m_{0}\left(i\right)$ < $m_{1}\left(i\right)$. Because the alternative hypothesis tests for an increase in the Pearson coefficient rather than for any difference in either direction, the test is directional.

**Figure A1:**
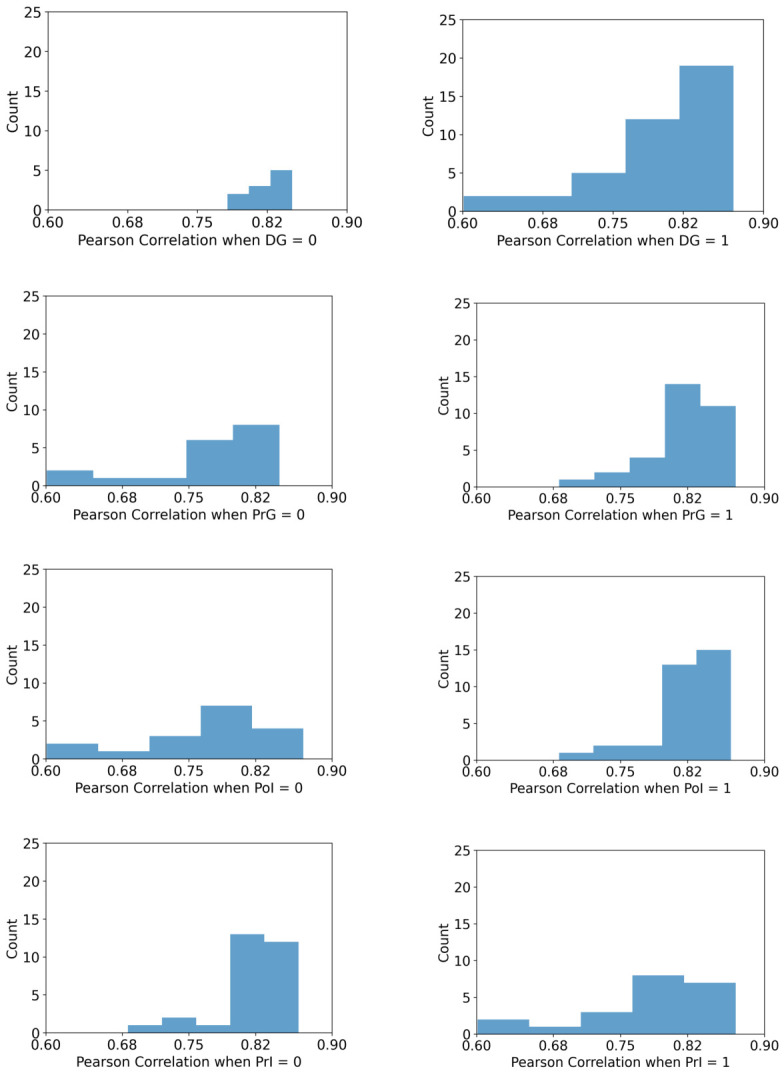
Distribution of Pearson correlation coefficients for DG, PrG, PoI and PrI binary properties. Each row corresponds to a different property (from top to bottom: DG, PrG, PoI, and PrI). For each row, the left column shows samples where the property is absent (0), while the right column shows samples where the property is present (1).

**Figure A2:**
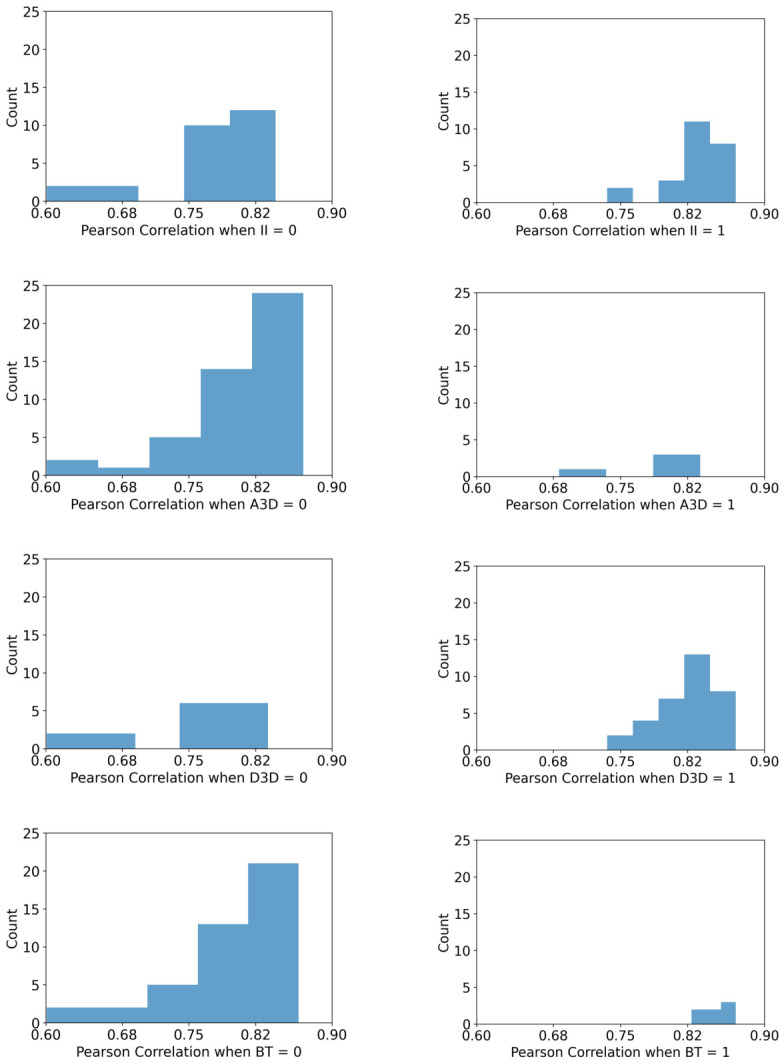
Distribution of Pearson correlation coefficients for the II, A3D, D3D, and BT binary properties. Each row corresponds to a different property (from top to bottom: II, A3D, D3D, and BT). For each row, the left column shows samples where the property is absent (0), while the right column shows samples where the property is present (1).

In summary, the Student’s *t*-test is valid when data have the following properties [10]: (1) The variable is a continuous numerical value, which is the case for the pIC50. (2) The values in each group come from populations that are approximately normally distributed. Appendix A incorporates images of the distributions, which, in general, have this desired behaviour (Figure A1 and Figure A2). (3) Measurements should not influence each other. Clearly, models are independent. Finally, (4) the size of sets might not be extremely imbalanced. Table 1 lists the size of each group and the balance ratio. We realise properties **DG**, **A3D** and **BT** have to be excluded from the Student’s *t*-test due to the imbalance problem (considering a balanced property has a ratio larger than 1/3).

Table 2 shows the results of the Student’s *t*-test given our hypothesis test of means.

The test confirms that the important binary properties for having a high Pearson coefficient are the protein (or part of the protein) being a graph (**PrG**), being the pocket and the interaction part of the input (**PoI** and **II**) and incorporating the 3D information of the atoms into the model (**D3D**). These are the properties that achieved $H_{B}\left(i\right)=1$. The pValue(i) confirms the result of testing $H_{B}\left(i\right)$, since a pValue(i) lower than 0.005 indicates that $H_{B}\left(i\right)$ is accepted.

The *t*-test shows that the introduction of the whole protein, **PrI**, is not necessary, although the introduction of the pocket, **PoI**, is necessary. The combination of these properties suggests that introducing the drug and the pocket as a graph representation—rather than the whole protein—is a good choice, as it provides sufficient structural knowledge for accurate binding affinity prediction while maintaining a relatively simple model architecture.

When deciding between introducing **A3D** or **D3D** (they are complementary), the *t*-test shows that the 3D data have to be introduced but not as (x, y, z) information. We believe this is because the specific positions of the atoms make the model not independent of rotations. This option forces us to discard **A3D** although this is not deduced in the *t*-test.

As properties **DG** and **BT** are imbalanced and a *t*-test was not feasible, they are directly analysed given the information in Table 5. We consider that: (1) There is no method that represents the protein (or part of the protein) as a graph (**PrG** = 1) and the drug as a vector (**DG** = 0), since it would make a more complex architecture. (2) The *t*-test considers it important that **PrG** = 1. (3) The six better models incorporate **DG** = 1. Then, we conclude **DG** = 1 is an important property. Finally, we also include the type of bonds between graphs, **BT** = 1, since this property is scarce but appears in four out of the top five best models.

### 2.2. Analysis of the Independence of Variables

The Chi-square test of independence is a non-parametric statistical method used to assess whether two categorical variables are associated or occur independently of one another. Its rationale is based on comparing the observed joint frequency distribution of the variables with the distribution expected under the assumption of independence. If the variables are independent, the observed frequencies in each category should closely match those expected from the product of their marginal distributions. This test returns a *p*-value, which quantifies the probability of observing a discrepancy between observed and expected frequencies as large as the one obtained, assuming the null hypothesis of independence is true. A small *p*-value indicates evidence against independence, suggesting an association between the variables.

Table 3 shows the *p*-value obtained by the Chi-square test for the assumption of independence between variables. If a confidence of 0.02 is considered, the table shows the dependence between four properties: **PrG**, **PoI**, **PrI** and **II**. Properties **PoI**, **PrI** and **II** belong to the second category that considers the input of the model. This result is not surprising, since in most cases, researchers decide to include some information in the input but not all of them. The dependence on **PrG** appears due to the volume of the data. If the protein is introduced as a graph, usually it is only incorporated as the pocket but not the whole protein. Moreover, there is also a dependence between **II** and **D3D**. Researchers who are aware of the importance of the ligand–protein interactions also are aware of the importance of these interactions being independent of the rotations and translations of the compounds. For this reason, few models introduce the interactions but impose the 3D positions of the atoms. As previously reported, there is not any model with the combination **DG** = 0 and **PrG** = 1, which creates strong dependence validated by the *p*-value = 0.0183. Finally, there is a tendency toward models with **D3D** = 0 (no information on the distances between atoms) having **PrI** = 1 (the protein is introduced). This configuration seems to be logical in reducing the space and computational cost and is reported by *p*-value = 0.0146.

It is important to emphasize that identifying statistical dependence among variables does not contradict the assessment of their individual importance. Tests of independence, such as the Chi-square test, evaluate whether variables are associated in a general sense, without specifying the nature or strength of their effect on the outcome of interest. In contrast, significance tests such as the Student’s *t*-test assess whether differences in group means are statistically meaningful, thereby providing insight into the contribution of each variable to the experimental response. Consequently, a set of variables may exhibit statistical dependence (i.e., **PrG**, **PoI**, **PrI** and **II**) while only a subset demonstrates a significant effect on the response variable (i.e., **PrG**, **PoI** and **II** are important although **PrI** is not). This distinction highlights that dependence reflects relationships among variables, whereas importance relates to their specific explanatory power within the context of the studied phenomenon.

## 3. Discussion

In recent years, several computational models have been presented to predict the binding affinity between proteins and ligands because it is an important task in the drug discovery field. The architectures presented are diverse, and for this reason, a FAIR comparison is needed, although difficult to achieve. PDBBind 2016 and CASF 2016 have become the de facto train set and test set, respectively. Moreover, these datasets incorporate the binding affinity through pIC50. For this reason, the Pearson coefficient of the predicted pIC50 and those in CASF 2016 is the usual measure of the quality of these models, although different hyper-parameters might be used.

Through the diversity of architectures of 50 models, this article extracts eight global properties and presents them in a general table. Moreover, in another table, it lists the Pearson coefficient and the RMSE published in several articles obtained by these models together with the references of the models and the references from which the Pearson coefficient and the RMSE have been extracted. Given these two tables and using statistical tests, three principal conclusions are deduced:

- The five most important properties that the models must have to achieve the highest Pearson coefficient are:
  (1) The model introduces the drug as a graph.
  (2) The pocket has to be selected (the atoms and the bonds of the protein that are “close to” the drug) and introduced into the model.
  (3) The model introduces the interactions between the protein and the drug.
  (4) The model uses the 3D information on the atoms. Nevertheless, the 3D information must be invariant to rotation and translation. For this reason, the distance between the atoms must be introduced into the model and not the specific 3D position.
  (5) The model uses the type of chemical bonds.
- Most of the models do not introduce the protein, the pocket and the interactions between pocket and protein at the same time. The authors assume this is due to the need to reduce the volume of the data and the belief in the redundancy of this data.
- The protein is not entirely introduced as a graph. It is introduced only by the pocket as a graph or the whole protein as a vector or string. The authors believe this is because the graph of a protein is too large to be processed or because of runtime restrictions.

The fact that the models have not been retrained with unified training protocols could be seen as a weakness of the Experimental Section. Clearly, using the reported Pearson coefficient in some scientific journals introduces hyper-parameter tuning bias. Note that inconsistencies in the training and testing sets might not occur since only the models tested using PDBBind 2016 and CASF 2016 as training and testing sets, respectively, have been selected. Nevertheless, it is supposed that authors present the best results, which means that the unified training protocol is to use the combination of hyper-parameters that achieve the higher Pearson coefficient instead of using exactly the same set of hyper-parameters, which is unachievable considering the huge differences in the 50 architectures.

The conclusions have been obtained only with the Pearson coefficient. The RMSE is also an interesting metric with which to evaluate the quality of the binding affinity. Nevertheless, Table 4 shows a clear dependency between these to variables, and thus, we consider the analysis of one of them enough.

## 4. Materials and Methods

### 4.1. Binding Affinity Prediction Based on Machine Learning

**Figure 1:**
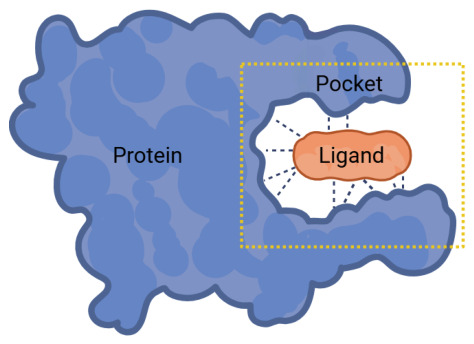
Graphical example of a protein–ligand interaction. The large outer shape represents the protein, which contains an internal cavity known as the binding pocket (outlined by the dotted rectangle). The smaller shape inside the pocket corresponds to the ligand, which interacts with surrounding residues through multiple contacts (shown as connecting lines).

This section provides a brief description of the basic concepts required for the paper, including binding affinity, the pocket, and drug–protein interaction. To enhance comprehension, these concepts are shown graphically in Figure 1.

Binding affinity describes how tightly two molecules bind to one another. In biology and pharmacology, it generally refers to the interaction between a drug, also called a ligand, and a protein. Usually, the drug is a much smaller chemical compound than the protein. For example, the mean number of atoms in a protein may be around 5000, while a drug may contain approximately 100 atoms or less, making the drug roughly 100 times smaller [2]. If a drug binds strongly to its target protein, a smaller dose is generally required to achieve a therapeutic effect. In contrast, weak binding may render the drug ineffective or require higher concentrations. Consequently, high binding affinity is often a goal in drug design, and thus the machine learning methods aim to discover new drugs whose predicted affinities are among the highest (in some specific situations, moderate binding is preferred to avoid side effects or allow reversibility).

A pocket is a small cavity or groove on the protein surface where drugs can bind. It is usually formed by the 3D arrangement of amino acid residues that create a shape complementary to the ligand. The shape, size, and chemistry of the pocket are very important in determining the binding affinity because it determines whether a drug fits (also called shape complementarity). Moreover, it also determines how strongly it binds (considering hydrogen bonds, hydrophobic interactions, and charge complementarity).

Protein–ligand interaction usually means how the drug molecule (or ligand) interacts with the protein’s binding pocket. The binding affinity is supposed to depend largely on the protein–ligand interaction. This is because the protein–ligand interaction is really about the molecular forces that “glue” the drug to the protein site. Inside the pocket, ligands are stabilized by non-covalent interactions, such as hydrogen bonds, hydrophobic interactions, electrostatic (ionic) interactions, van der Waals forces or π-π stacking and cation-*π* interactions. Each of these interactions contributes to the overall binding affinity.

### 4.2. Practical Experiments on Binding Affinity Prediction

PDBBind 2016 is a widely used benchmark database that curates experimentally measured binding affinities for protein–ligand complexes with available 3D structures from the Protein Data Bank. It is organized into hierarchical subsets, including the General Set, the higher-quality Refined Set, and the Core Set, which contains 285 diverse and non-redundant complexes and is commonly used for unbiased model evaluation. Moreover, CASF 2016 (Comparative Assessment of Scoring Functions) is a standardized benchmarking framework built on the PDBBind 2016 Core Set and defines consistent evaluation protocols for binding affinity prediction. It assesses model performance across multiple tasks, including scoring, ranking, docking, and virtual screening power, with scoring power typically quantified using the Pearson correlation coefficient $R_{p}$ between predicted and experimental affinities.

The scientific community generally considers it appropriate to compare computational models using the Pearson correlation coefficient between predicted and experimental binding affinities, with the publicly available PDBBind 2016 and CASF 2016 datasets used as the training and test sets, respectively. This is because these datasets are considered FAIR (Findable, Accessible, Interoperable, and Reusable). This means that different methods are evaluated under identical, transparent, and reproducible conditions, so that performance differences reflect the models themselves rather than experimental artifacts. For this reason, together, PDBBind 2016 and CASF-2016 provide a rigorous and widely adopted foundation for the development and comparison of protein–ligand binding affinity prediction models. Nevertheless, the architecture of the models is diverse and the hyper-parameter tuning has not been discovered; for this reason, we have to be aware of the difficulty of comparing these 50 models.

Table 4 shows the Pearson coefficient $R_{p}$ and the RMSE (Rooted Mean Square Error) obtained by the models when predicting the binding affinity (measured by pIC50). These values have been extracted from several papers and we checked that when a result of one model appears in more than one article, the numbers coincide. In all of them, the training set was the PDBBind 2016 database, and the test set was the CASF 2016 database. Nevertheless, these architectures are completely different, and it is difficult to standardise the hyper-parameters. The authors declare that they present the results obtained by the most favourable hyper-parameter configuration. The last column, *Data Reference*, indicates the reference from which these results have been extracted. In some cases, the reference for the model (second column) differs from the data reference (last column). This occurs because the original articles did not evaluate their models on binding affinity prediction using the specific training and test sets PDBBind 2016 and CASF 2016, respectively. Some of the models have been specifically designed for binding affinity prediction, whereas others were originally designed for broader chemical property prediction and were later applied to the binding affinity task by subsequent authors.

The models were published within the period from 2017 to 2025 and are ordered by decreasing Pearson coefficient, which ranges from 0.86 to 0.60. Notably, more than half of the models achieved a Pearson coefficient greater than 0.80. Moreover, in almost all cases, when the Pearson coefficient decreases, the RMSE increases. Finally, the RMSE of three models is not reported in any article, since some of them only report the Pearson coefficient.

**Table 4:** Comparison of various models by the Pearson correlation coefficient $R_{p}$ and the RMSE (Rooted Mean Square Error), displayed from the best to the worst $R_{p}$.

Model	Ref.	Year	$R_{p}$	RMSE	Data Ref.
CheapNET	[11]	2025	0.870	1.107	[11]
saCNN	[12]	2021	0.865	1.117	[13]
TopBP	[14]	2018	0.861	1.650	[15]
egGNN	[16]	2021	0.860	1.122	[16]
SS–GNN	[15]	2023	0.853	1.181	[15]
MP-GNN	[17]	2022	0.851	-	[17]
DAAP	[18]	2025	0.845	1.196	[18]
Mol-PSI	[19]	2022	0.844	1.278	[15]
DCML	[20]	2022	0.843	1.255	[15]
CAPLA	[21]	2023	0.841	1.206	[17]
GIGN	[22]	2023	0.840	1.190	[17]
PerSpect ML	[23]	2021	0.840	1.724	[15]
FPRC	[24]	2021	0.838	-	[17]
AGL-Score	[25]	2019	0.833	1.733	[25]
SableBind	[26]	2025	0.832	1.205	[26]
HPC/HWPC	[27]	2022	0.831	1.307	[15]
CurvAGN	[28]	2023	0.830	1.217	[28]
DEAttentionDTA	[29]	2024	0.827	1.266	[11]
PLEC	[30]	2019	0.826	-	[17]
DeepAtom	[31]	2019	0.825	1.232	[13]
DG-GL	[32]	2019	0.825	1.767	[32]
PLANET	[33]	2024	0.824	1.247	[33]
LGN-GIN	[34]	2024	0.822	1.333	[34]
IGN	[35]	2021	0.821	1.269	[11]
K_Deep_	[36]	2018	0.820	1.270	[15]
EGNN	[37]	2021	0.816	1.289	[11]
OnionNet	[38]	2019	0.816	1.278	[15]
Fusion-Score	[13]	2021	0.815	1.300	[13]
ELGN	[39]	2024	0.810	1.285	[28]
TNET-BP	[40]	2017	0.810	1.340	[40]
FAST	[41]	2021	0.810	1.308	[41]
GAABind	[42]	2024	0.803	1.297	[11]
SIGN	[43]	2021	0.797	1.316	[28]
LGN-GAT	[34]	2024	0.794	1.424	[34]
SchNet	[44]	2017	0.787	1.390	[11]
DeepDTAF	[45]	2021	0.785	1.357	[45]
AttentionSiteDTI	[46]	2022	0.784	1.352	[11]
Pafnucy	[47]	2018	0.780	1.420	[15]
PotentialNET	[48]	2018	0.772	1.503	[11]
CMPNN	[49]	2020	0.765	1.408	[28]
LGN-GTN	[34]	2024	0.767	1.481	[34]
GraphDTA-GAT-GCN	[50]	2021	0.754	1.434	[11]
MGraphDTA	[51]	2022	0.753	1.439	[11]
DimeNet	[52]	2021	0.752	1.453	[28]
MAT	[53]	2020	0.747	1.457	[28]
GNN-DTI	[54]	2019	0.736	1.492	[28]
SGCN	[55]	2020	0.686	1.583	[28]
GraphDTA-GIN	[50]	2021	0.667	1.640	[28]
GraphDTA-GCN	[50]	2021	0.613	1.735	[28]
GraphDTA-GAT	[50]	2021	0.601	1.765	[28]

### 4.3. Structural Properties of the Binding Affinity Methods

This section shows a new taxonomy for organising computational models for binding affinity prediction based on eight binary properties (exist/absent). They describe aspects of the structure of the compound, including the protein, the drug, and their interactions. They are grouped into three categories.

The first category considers how the drug and the protein are represented. Basically, two types of representation have been proposed: the ones that generate a vector of properties, such as fingerprints, and the ones that generate attributed graphs, in which nodes are atoms or residues and edges are bonds or energies between them. There are two properties in this group:

- **DG**: The **D**rug is represented as an attributed **G**raph. Other alternatives are to represent the drug as a string (SMILES), molecular fingerprints (Morgan, ECFP, MACCS keys) or Physicochemical Descriptor Vectors (LogP, TPSA). In all the models, the whole drug, usually much smaller than the protein, is included.
- **PrG**: The **Pr**otein or a part of the protein is represented as an attributed **G**raph. Some of the models do not include the whole protein but only the pocket. The next category is used to distinguish between both cases.

The second category considers the model’s input, and more specifically the representation of the protein, since all models incorporate the drug as part of the input. In this category, three properties are considered:

- **PrI**: The whole **Pr**otein is part of the **I**nput. As in **PrG**, some models only include the pocket.
- **PoI**: The **Po**cket is part of the **I**nput. Some models specifically incorporate the pocket, although they can also include the whole protein or not. The existence of this property is not contradictory to the existence of **PrI**. Nevertheless, if both exist, some redundant information is introduced into the model.
- **II**: **I**nteraction between the protein and drug is part of the **I**nput. Some models deduce the interactions between the protein and drug, such as an estimation of the non-covalent bonds, and then consider them in the model.

Finally, the third category consists of three properties related to the atoms and the synergies between them. More specifically, they are related to the 3D position of the atoms and the type of bonds that appear.

- **A3D**: The **A**tom’s **3D** positions are considered. The model introduces the 3D position of the atom, usually obtained by the SDF and PDB files or some chemical computational programs. This knowledge is written in the node attributes.
- **D3D**: **D**ata on **3D** positions is considered, although it is not part of the node attributes. Some distances or functions are computed using the 3D position, but the 3D information itself is not stored in the node attributes. Therefore, **D3D** and **A3D** are mutually exclusive.
- **BT**: **B**ond **T**ypes are considered as attributes of the graph edges. In the same way as for the 3D position, this information is extracted from the SDF and PDB files or computed in some chemistry programs. In contrast, some models deduce the existence of a pair-of-atoms relation, and this information is structured as an edge between nodes. However, edges do not have attributes. A common option is to impose an edge if the distance between atoms is smaller than a given threshold, for example, to 5 Å.

Table 5 presents the same set of 50 models used for drug–protein binding affinity prediction shown in Table 4, together with their eight binary properties (where 1 indicates presence and 0 indicates absence).

In general, the computational cost and memory requirements increase when either the drug or the protein is represented as an attributed graph. Considering that the proteins are typically much larger than the drugs, several models consider only representing the drug as a graph. Others represent only the binding side as a graph, excluding the remaining part of the protein. As a general rule, the slowest and more memory-demanding models are those that represent both the drug and the whole protein as a graph (**DG** = 1, **PrG** = 1 and **PrI** = 1). These models, in Table 5, include CheapNET, TopBP, SableBind, Fusion-Score, PotentialNET and CMPNN. Among them, TopBP also incorporates the pocket (**PoI** = 1), which means that three attributed graphs are included in the model.

In contrast, the presence or absence of properties in the third category (**A3D**, **D3D** and **BT**) has relatively little impact on computational power and/or memory demand.

Only the five models—DAAP, CAPLA, DEAttentionDTA, PLEC and OnionNet—introduce the pocket as a non-graph representation (**PrG** = 0 and **PoI** = 1), such as a vector or a fingerprint. Among them, only DAAP and PLEC also model drug–protein interactions as a non-graph (**PrG** = 0, **PoI** = 1 and **II** = 1). The other three models do not consider interactions (**PrG** = 0, **PoI** = 1 and **II** = 0).

By definition of the properties, it is not possible for both **A3D** = 1 and **D3D** = 1 to occur simultaneously. Moreover, there is no relation between the presence or absence of these properties and whether the drug or protein is represented as an attributed graph.

Finally, seven models explicitly account for the types of bonds: CheapNET, TopBP, egGNN, SS-GNN, GIGN, SableBind and PLANET. All these models represent both the drug and the protein as graphs (i.e., **BT** = 1, **DG** = 1 and **PrG** = 1).

**Table 5:** Models in Table 4 are also displayed from the best to the worst $R_{p}$. Further, 1 or 0 symbolise the existence or non-existence of the property. **DG**: The **D**rug is represented as an attributed **G**raph. **PrG**: The **Pr**otein is represented as an attributed **G**raph. **PoI**: The **Po**cket is part of the **I**nput. **PrI**: The **Pr**otein is part of the **I**nput. **II**: **I**nteraction between Protein and Drug is part of the **I**nput. **A3D**: **A**tom **3D** positions are considerable. **D3D**: **D**ata on **3D** positions are considerable. **BT**: **B**ond **T**ypes are considerable.

	Representation		Model Input			Struct. Features			
Model	Ref.	DG	PrG	PoI	PrI	II	A3D	D3D	BT
CheapNET	[11]	1	1	0	1	1	0	1	1
saCNN	[12]	1	1	1	0	1	0	1	0
TopBP	[14]	1	1	1	1	1	0	1	1
egGNN	[16]	1	1	1	0	1	0	1	1
SS-GNN	[15]	1	1	1	0	1	0	1	1
MP-GNN	[17]	1	1	1	0	1	0	1	0
DAAP	[18]	0	0	1	1	1	0	1	0
Mol-PSI	[19]	1	1	1	0	1	0	1	0
DCML	[20]	1	1	1	0	1	0	1	0
CAPLA	[21]	0	0	1	1	0	0	1	0
GIGN	[22]	1	1	1	0	1	0	1	1
PerSpect ML	[23]	1	1	1	0	0	0	1	0
FPRC	[24]	1	1	1	0	1	0	1	0
AGL-Score	[25]	1	1	1	0	1	1	0	0
SableBind	[26]	1	1	0	1	0	0	0	1
HPC/HWPC	[27]	1	1	1	0	1	0	1	0
CurvAGN	[28]	1	1	1	0	1	0	1	0
DEAttentionDTA	[29]	0	0	1	1	0	0	0	0
PLEC	[30]	0	0	1	0	1	0	0	0
DeepAtom	[31]	0	0	0	0	0	0	1	0
DG-GL	[32]	1	1	1	0	1	0	1	0
PLANET	[33]	1	1	1	0	1	0	1	1
LGN-GIN	[34]	1	1	1	0	1	0	1	0
IGN	[35]	1	1	1	0	1	0	1	0
K_Deep_	[36]	0	0	0	1	0	0	1	0
EGNN	[37]	1	1	1	0	0	0	1	0
OnionNet	[38]	0	0	1	0	0	0	1	0
Fusion-Score	[13]	1	1	0	1	0	1	0	0
ELGN	[39]	1	1	1	0	1	0	1	0
TNET-BP	[40]	0	0	0	1	0	0	1	0
FAST	[41]	1	1	1	0	0	1	0	0
GAABind	[42]	1	1	1	0	0	0	1	0
SIGN	[43]	1	1	1	0	1	0	1	0
LGN-GAT	[34]	1	1	1	0	1	0	1	0
SchNet	[44]	1	0	0	1	0	0	1	0
DeepDTAF	[45]	0	0	1	1	0	0	0	0
AttentionSiteDTI	[46]	1	1	1	0	0	0	1	0
Pafnucy	[47]	0	0	0	1	0	0	1	0
PotentialNET	[48]	1	1	0	1	0	0	1	0
CMPNN	[49]	1	1	0	1	0	0	0	0
GraphDTA-GAT-GCN	[50]	1	0	0	1	0	0	0	0
LGN-GTN	[34]	1	1	1	0	1	0	1	0
MGraphDTA	[51]	1	0	0	1	0	0	0	0
DimeNet	[52]	1	0	0	1	0	0	0	0
MAT	[53]	1	0	0	1	0	0	0	0
GNN-DTI	[54]	1	1	1	0	1	0	1	0
SGCN	[55]	1	1	1	0	0	1	0	0
GraphDTA-GIN	[50]	1	0	0	1	0	0	0	0
GraphDTA-GCN	[50]	1	0	0	1	0	0	0	0
GraphDTA-GAT	[50]	1	0	0	1	0	0	0	0

## 5. Conclusions

The aim of this paper is not to present a new survey of computational models for binding affinity prediction, because other ones have been presented, but a statistical analysis of the main properties that the best models have, with the aim of promoting better models in future research.

The final conclusion **extracted from the statistical analysis** is that future models have to incorporate:

- Structural information of the pocket and the ligand.
- The chemical relations generated between the protein and the ligand.
- 3D information independent of rotations and translations.

The property “drug being introduced as a graph” is not incorporated into the *t*-test due to the imbalance problem. The best and newest models incorporate this property, and only ten models represent the ligand as a vector instead of a graph. The final conclusion **extracted from the research analysis** is that future models have to incorporate:

- The drug as an attributed graph.

Future work is going to involve the development of a new architecture based on the main properties listed above, and evaluate it using the PDBbind/CASF 2016 datasets to experimentally demonstrate its usefulness.

## Figures and Tables

**Table 1:** Size of the groups split by having the property (first column) and not having the property (second column). The last column shows the balance ratio measured as the minimum size of both sets divided by the maximum size of them. In bold, properties with a balance ratio smaller than 1/3.

Property	1	0	Min/Max
**DG**	40	10	**0.25**
**PrG**	32	18	0.56
**PoI**	33	17	0.51
**PrI**	21	29	0.72
**II**	24	26	0.92
**A3D**	4	46	**0.08**
**D3D**	34	16	0.47
**BT**	7	43	**0.16**

**Table 2:** Hypothesis test of means given the data in Tables 4 and 5.

Property	*i*	$H_{B}\left(i\right)$	pValue(i)
**PrG**	2	1	0.0029
**PoI**	3	1	0.0005
**PrI**	4	0	0.9922
**II**	5	1	0.0003
**D3D**	7	1	0.0000

**Table 3:** *p*-values computed by the Chi-square test to check the independence assumption. In bold are the smaller values, which means that the variables are considered dependent (confidence 0.02).

	DG	PrG	PoI	PrI	II	A3D	D3D	BT
**DG**	0	**0.0183**	0.9665	1.0000	1.0000	0.7703	0.9963	0.8672
**PrG**		0	**0.0039**	**0.0002**	**0.0016**	0.6652	0.0666	0.7258
**PoI**			0	**0.0000**	**0.0004**	0.9126	0.0365	0.9925
**PrI**				0	**0.0009**	0.8471	**0.0146**	0.9998
**II**					0	0.7945	**0.0078**	0.7228
**A3D**						0	1.0000	0.8455
**D3D**							0	0.9211
**BT**								0

## Data Availability

The code to generate Table 2 and Table 3 is available at: https://github.com/FrancescSerratosa/Structural-Knowledge-Binding-Affinity, accessed on 3 June 2026.
